# Acute high-intensity interval exercise improves food-related cognition in young adults with obesity: An ERP study

**DOI:** 10.1016/j.ijchp.2023.100430

**Published:** 2023-12-20

**Authors:** Chun Xie, Brandon L. Alderman, Fanying Meng, Ying-Chu Chen, Yu-Kai Chang, Kun Wang

**Affiliations:** a Department of Physical Education, Shanghai Jiao Tong University, Shanghai, China; b Department of Kinesiology and Health, Center of Alcohol and Substance Use Studies, Rutgers University – New Brunswick, New Brunswick, NJ, United States; c Institute of Physical Education, Huzhou University, Huzhou, China; d Department of Physical Education and Sport Sciences, National Taiwan Normal University, Taipei, Taiwan; e Institute for Research Excellence in Learning Science, National Taiwan Normal University, Taipei, Taiwan; f Social Emotional Education and Development Center, National Taiwan Normal University, Taipei, Taiwan

**Keywords:** High-intensity interval exercise (HIIE), Food-related cognition, Obesity, P3, LPP

## Abstract

*Purpose* Cognitive function, particularly food-related cognition, is critical for maintaining a healthy weight and preventing the acceleration of obesity. High-Intensity Interval Exercise (HIIE) is an increasingly popular form of exercise and has been shown to improve physical fitness and cognitive function. However, there is limited research on the effects and underlying mechanisms of HIIE on general and food-related cognition among adults with obesity. The aim of the current study was to examine the influence of a single bout of HIIE on food-related cognition among young adults with obesity.

*Methods* Fifteen young men with obesity (BMI = 33.88 ± 4.22, age = 24.60 ± 5.29 years) were recruited. Participants took part in a HIIE condition consisting of 30 minutes of stationary cycle exercise (5-min warm-up, 20-min HIIE and 5-min cool down), and a control session consisting of a time and attention-matched period of sedentary rest in a counterbalanced order. Behavioral (reaction time and accuracy) and event-related potential measures (P3 and the late positive potential, LPP) elicited during a food-related Flanker task were measured after the HIIE and control session.

*Results* Shorter response times were observed following HIIE, regardless of congruency or picture type, with no change in accuracy. Increased P3 and LPP amplitudes were observed following HIIE relative to the control session.

*Conclusion* The findings suggest a single bout of HIIE has a beneficial effect on general and food-related cognition among young adults with obesity, with increased recruitment of cognitive resources to support cognitive control. Future research is warranted to examine the dose-response relationship between acute bouts or longer participation in HIIE on food-related cognition in obesity.

## Introduction

Obesity is a major public health concern worldwide, and is associated with increased risk of type II diabetes, cancers, and cardiovascular disease (Guh et al., 2009; Jensen et al., 2014; LeBlanc et al., 2018) as well as cognitive decline (Prickett et al., 2015; Smith et al., 2011). A number of cross-sectional, longitudinal and meta-analytic studies have demonstrated that obesity is not only linked to a decrease in general cognitive function (Prickett et al., 2015; Smith et al., 2011) but also to impairments in inhibitory control (Kamijo et al., 2014; Yang et al., 2018), a core component of executive function (Miyake et al., 2000). Surrounded by an obesogenic environment with plenty of high-calorie and easily accessible foods (Hall, 2018), cognitive and inhibitory control is vital for restraining the impulse to consume highly palatable foods to maintain a healthy weight and prevent the acceleration of obesity (Bailey et al., 2021; Lavagnino et al., 2016). However, numerous studies have shown that obese individuals perform more poorly than their healthy-weight counterparts in general and food-specific cognition, including inhibitory control (Kamijo et al., 2014; Lavagnino et al., 2016; Prickett et al., 2015; Reinert et al., 2013; Smith et al., 2011). Systematic reviews and meta-analyses have also demonstrated that obesity is associated with cognitive deficits across the lifespan in nearly all domains, including complex attention (Prickett et al., 2015; Smith et al., 2011), and high-order cognitive-executive function, which includes inhibition and decision-making/reward processes (Lavagnino et al., 2016; Li et al., 2023; Reinert et al., 2013; Vainik et al., 2013; Yang et al., 2018). Moreover, individuals with obesity have shown alterations in food-related cognition, using tasks that tap food-related inhibitory control, attention allocation, and reward responsiveness (Castellanos et al., 2009; García-García et al., 2020; García-García et al., 2013; Hagan et al., 2020; Kenny, 2011; Lavagnino et al., 2016; Leng et al., 2022; Li et al., 2023; Wang et al., 2022). Therefore, targeting both general and food-related cognition may help with food selection, portion control, and ultimately, serve as treatment targets for interventions aiming to prevent the acceleration of obesity.

Numerous empirical studies have provided robust evidence that acute bouts of participation in aerobic exercise can improve cognitive and executive function (Chang et al., 2012; Chu et al., 2015; Drollette et al., 2014; Etnier et al., 1997; Faria et al., 2022; Gusatovic et al., 2022; Hsieh et al., 2018; Kao et al., 2022; Levin et al., 2021; Ren et al., 2023). This effect has also been extended to obese populations (Chang et al., 2017; Logan et al., 2021; Napoli et al., 2014; Peven et al., 2020; Secor, 2020). However, a growing body of literature suggests that high-intensity interval exercise (HIIE) is as effective or perhaps more effective than traditional moderate-intensity aerobic exercise in facilitating cognitive function and inhibitory control among healthy-weight adults (Ai et al., 2021; Kao et al., 2018; Kao et al., 2017; Tian et al., 2021). HIIE was initially shown to improve cardiorespiratory fitness and body composition (Alves et al., 2017; Delgado-Floody et al., 2019; Huang et al., 2022; Wewege et al., 2017) but more recent research has also extended the benefits of HIIE to cognitive function (Ai et al., 2021; Alves et al., 2014; Hsieh et al., 2021; Kao et al., 2018; Kao et al., 2017; Roig-Hierro et al., 2023; Tian et al., 2021; Tsai et al., 2021; Tsukamoto et al., 2016). However, whether the acute cognitive benefits of HIIE extend to obese individuals has yet to be fully explored. Quintero et al. (2018) conducted a preliminary study on the cognitive effects of acute HIIE among an overweight population. They found initial evidence that acute HIIE could improve cognitive function in overweight individuals. Previous research from our lab also demonstrated faster response times in congruent and incongruent conditions of a Flanker task following acute HIIE when compared with task performance following a sedentary control session among 16 obese young adult men (Xie et al., 2020). We found similar improvement in a Stroop task following HIIE (Zhang et al., 2022), suggesting that a single bout of HIIE may facilitate cognitive function, and specifically inhibitory control among individuals with overweight and obesity.

Though previous studies have provided valuable evidence supporting the beneficial effect of HIIE on general cognitive function among obese individuals, little attention has been given to the impact of HIIE on food-related cognition. However, several recent studies have focused on the effects of aerobic exercise on food-related cognition. Bailey et al. (2021) reported that an acute bout of aerobic exercise performed at 70% VO_2max_ improves food-related inhibitory control. Zhang et al. (2020) similarly documented that a single bout of coordinative exercise resulted in enhanced food-related inhibitory control in obese adolescents. Some research suggests decreased neural response to a food-related reward or attention task following an acute bout of aerobic exercise in obese individuals (Fearnbach et al., 2016; Fearnbach et al., 2017; Hanlon et al., 2012). Others studies have focused on the effects of acute HIIE on appetite or food intake. For example, Hu et al. (2022) conducted a meta-analysis and reported that acute HIIE suppresses appetite and energy intake immediately post exercise (Hu et al., 2022). Miguet et al. (2018) documented that a single session of HIIE decreases fat and sweet reward preference as well as subsequent energy intake among adolescents with obesity. Considering the potentially important role of food-related cognition in weight management, further investigation of the influence of HIIE on food-related cognition is warranted.

Although there is growing body of literature supporting the effects of HIIE on cognition (Ai et al., 2021; Alves et al., 2014; Tsukamoto et al., 2016), the underlying mechanisms of HIIE on cognitive function remains unclear (Drollette et al., 2022; Kao et al., 2018; Kao et al., 2017; Tsai et al., 2021). Event-related potentials (ERPs), derived from the ongoing electroencephalogram (EEG), are a relatively non-invasive high temporal resolution methodology that can be used to reveal precise neurocognitive processes. ERPs have been used to examine neurocognitive mechanisms underlying the effects of acute exercise on general cognitive function (Chu et al., 2015; Fearnbach et al., 2016; Fearnbach et al., 2017; Hanlon et al., 2012; Kao et al., 2022; Levin et al., 2021; Ludyga et al., 2017; Themanson & Hillman, 2006) and food-related cognition among individuals with overweight and obesity (Chu et al., 2015; Fearnbach et al., 2016; Fearnbach et al., 2017; Hanlon et al., 2012; Kao et al., 2022; Levin et al., 2021; Ludyga et al., 2017; Themanson & Hillman, 2006). The P3 component is one of the mostly frequently examined ERP components in this area (Drollette et al., 2022; Kao et al., 2018; Kao et al., 2017; C. L. Tsai et al., 2021), although the late positive potential (LPP), a response to stimulus significance that has been defined in terms of the activation of appetitive and aversive motivational systems, has been studied less frequently using food-related cognitive tasks (Carbine et al., 2018; Hanlon et al., 2012). Our previous study examined this ERP component in obese populations (Xie et al., 2020), and we found that LPP amplitude, but not the P3, was facilitated after a 30-min session of HIIE, suggesting an increased attentional allocation and cognitive control after a single bout of HIIE. However, the underlying psychophysiological mechanisms underlying HIIE effects on food-related cognition among obese individuals remains understudied; therefore, it remains important to explore these neural processes following this increasingly popular mode of exercise.

The aims of the current study were to examine whether a single bout of HIIE could improve general and food-related cognition among young adults with obesity, and to examine the P3 and LPP components elicited by a food-related cognitive task to reveal underlying cognitive mechanisms. It was hypothesized that an acute bout of HIIE will have a beneficial effect on general and food-specific cognition and result in larger P3 and LPP amplitudes.

## Materials and methods

### Participants

We recruited fifteen young male adults aged 18 to 35 years from Shanghai. All participants met the following inclusion requirements: (a) body mass index (BMI) ≥ 28 kg/m^2^ (China obesity cutoff point) (He et al., 2015; Xu et al., 2015; Zhou, 2002); (b) normal or corrected-to-normal vision; (c) right hand dominance; (d) no contraindications to exercise according to the Physical Activity Readiness Questionnaire (Thomas et al., 1993). The following exclusion criteria were also used: (a) no prescription or weight loss drugs that may affect glucose and lipid metabolism within the previous month; (b) no endocrine and cardiovascular diseases (a Yes/No response); (d) no current mental illness (a Yes/No response); (e) no major illness (including malignant tumors, severe brain injuries, severe psychiatric illnesses, etc.). The initial study was confined to male participants due to the fact that prior research has indicated sex differences in food cravings, cognitive performance, and neural responses to food stimuli (Hallam et al., 2016; Ravichandran et al., 2021). All participants signed an informed consent approved by Shanghai University of Sport Ethics Committee (#102772019RT005). Demographic characteristics for the study sample are shown in Table 1.

**Table 1:** Demographic characteristics.

Variables	Mean ± SD
Age (years)	24.60 ± 5.29
Height (m)	1.77 ± 0.05
Weight (kg)	105.78 ± 14.04
BMI (kg/m^2^)	33.88 ± 4.22
Digital span	
Forward	14.40 ± 2.35
Backward	8.80 ± 3.67
Education level (years)	15.47 ± 2.23
VO_2peak_ (ml/kg/min)	33.63 ± 9.72
Basal metabolic rate (kJ/m^2^/h)	1112.72 ± 143.84
DEBQ	
Restrained eating	2.95 ± 0.56
External eating	2.39 ± 1.00
Emotional eating	3.47 ± 0.66

### Food-related image selection

60 high-calorie food images and 60 neutral pictures were selected from the Food Picture Database (Blechert et al., 2014). All pictures were matched for size, resolution, brightness, and background. Independent samples *t*-tests were used to compare the valence and arousal of the two-picture types. Results showed that the valence (49.24 ± 6.61 vs. 45.76 ± 7.18, *p* = 0.01), and arousal (33.45 ± 5.49 vs. 18.38 ± 6.85, *p* = 0.00) scores of the high-calorie food images were significantly higher than the neutral pictures, respectively. Some examples of the pictures are presented in Appendix A and pictures employed in the experiment from the food-pics database are listed in Appendix B.

### Food-related Flanker task

**Fig. 1:**
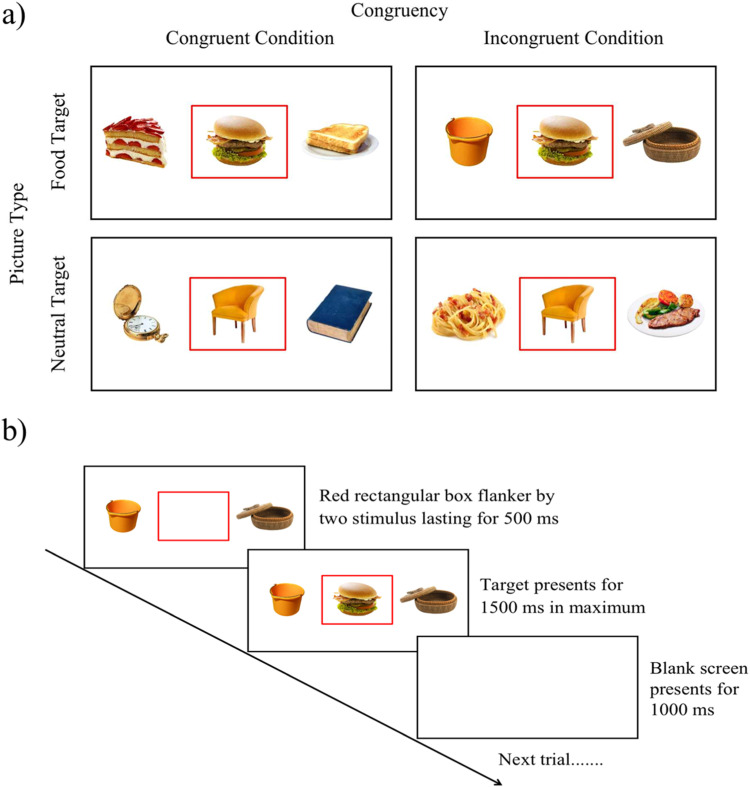
a) Example of the congruent and incongruent task conditions of the food-related Flanker task across both picture types (high-calorie food and neutral). b) The food-related Flanker task paradigm.

A computerized food-related Flanker task (Meule et al., 2012) was used to examine food-related cognitive control, which was implemented using E-Prime 2.0 (Psychological Software Tools, Pittsburgh, PA, United States). The paradigm was similar to the traditional Flanker task paradigm (Eriksen & Eriksen, 1974; Kamijo et al., 2014). For this task, participants were required to choose the target picture type (high-calorie food image vs. neutral image) in a red rectangular box in the middle and inhibit the congruent or incongruent images on both sides of the target image. An example of the congruency condition (congruent vs. incongruent) × picture type (high-calorie food image vs. neutral picture) is shown in Fig. 1**a**. Each trial began with a red rectangular box presented in the middle of the screen flanked by two stimulus pictures appearing on both sides of the box on a white background for 500 ms. Participants were required to gently place their right index finger on the number "5" key. Then, a target stimulus appeared in the red box, and participants were to press the number “4” key or number “6” key when the target stimulus in the red rectangle was a “neutral picture” or a “high-calorie food picture”, respectively. After pressing the response key, participants were told to return their finger to the number "5" key. The stimulus would disappear when the response key was pressed and each trial lasted for 1500 ms. Lastly, a blank screen was presented for 1000 ms (see Fig. 1**b**). The experiment had a practice block and three formal blocks of trials. The practice block consisted of 12 trials and participants were required to reach an accuracy of 80% before proceeding. If 80% was not reached, participants completed another practice block of 12 trials. Each formal block consisted of 120 trials containing all four conditions, and all stimulus images were presented in a random order.

### Electrophysiological recording and analysis

EEG signals were recorded by a 64-channel recording system using Ag/AgCl electrodes positioned according to the International 10-20 standard electrode system (Brain Products GmbH, Munich, Germany). EEG data were referenced online to the vertex electrode FCZ with AFz serving as the ground electrode and digitized continuously at 1000 Hz during data collection. An electrode was placed 1 cm outside of the right eye to record horizontal electrooculography (EOG) and 1 cm below the middle and lower orbit of the right eye to record vertical electrooculography (ECG). Electrode impedance was maintained at or less than 10 kΩ throughout the recording sessions.

ERP data were recorded using Brain Vision Recorder software and analyzed using Brain Vision Analyzer 2.0 software. Offline data were first re-referenced using the bilateral mastoids (TP9, TP10) as reference electrodes. Data were then high-pass filtered at 0.1 Hz and low-pass filtered at 30 Hz. ERP data were then segmented into epochs from 200 ms prior to stimulus onset to 1500 ms following stimulus onset, for 1700 ms time windows. All epochs were baseline adjusted using the 200 milliseconds pre-stimulus window and eye blinks were subsequently removed using independent components analysis (ICA). Channels were identified as bad if the fast average amplitude exceeded 100 microvolts (μV). The mean number of analyzed segments of high-calorie food picture target congruent condition, neutral picture target congruent condition, high-calorie food picture target incongruent condition as well as neutral picture target incongruent condition were 67.80 ± 21.81, 67.93 ± 22.84, 68.33 ± 22.20 and 68.70 ± 21.21, respectively. Analyses were conducted for the P3 and LPP area-average amplitudes, with time windows of 400-600 ms (P3) and 700-1100 ms (LPP) after stimulus onset at the midline parietal (Pz) electrode site (Ligeza et al., 2023).

### Experimental procedure

Participants were formally invited to the laboratory for two distinct sessions, with a HIIE session and a control session performed in a counterbalanced order separated by 3 days. Upon arrival, participants provided written informed consent and completed a demographic questionnaire. Intelligence (Digit Span test) (Wechsler, 1997) as well as the eating styles (Dutch Eating Behavior Questionnaire (DEBQ) (Van Strien et al., 1986) were then measured to characterize the study participants (Chi et al., 2021; Khandekar et al., 2023; Song et al., 2022; Zhang et al., 2022). Subsequently, height and weight were assessed for the determination of BMI (weight (kg)/height (m)^2^) and cardiorespiratory fitness (VO_2peak_) was assessed using the YMCA submaximal ergometer exercise test (Golding et al., 1989; Xie et al., 2020). This test is considered safe and well-tolerated by individuals with obesity, minimizing risks associated with maximal exercise testing and has been utilized in previous obesity-related studies (Chi et al., 2021; Song et al., 2016; Song et al., 2022).

Eligible participants took part in two sessions, and each session was conducted approximately 1 hour after eating. The HIIE session consisted of 30 minutes stationary cycle exercise (MONARK 894E, Sweden) consisting of a 5-min warm-up, 20-min formal HIIE exercise (10 cycles of 1-min 80-90% maximal Heart Rate (HR_max_) high-intensity exercise separated by 1-min 50-65% HR_max_ active relax), and a 5-min cool down. The control session consisted of a time and attention-matched sedentary period of seated rest, where subjects were asked to sit quietly in a chair and engage in active reading to ensure they were not in a completely closed-eye resting state. Heart Rate (assessed through a Polar heart rate monitor (FT1, Polar Electro Oly, Finland) and perceived exertion (RPE) (Borg, 1982) were recorded every 1-min during the exercise. Participants were asked to dry their scalp with a hairdryer immediately after exercise to minimize the influence of sweating on the EEG signal. Within 15 min following HIIE or control, the cognitive task and EEG recording were performed.

### Statistical analysis

The behavioral data were collected by E-Prime 2.0 software. The main indicators were reaction time and accuracy for congruent and incongruent task conditions of the food-related flanker task. For the behavioral data, MATLAB software was used to preprocess the original data, and outliers and extreme values were identified as average value plus or minus 3 times the standard deviation under each index and were eliminated.

The study used a within-subject design, with session, congruency and picture type as within-subject factors. For the behavioral data, a 2 (session: HIIE vs. control) × 2 (congruency: congruent vs. incongruent) × 2 (picture type: high-calorie food picture vs. neutral picture) repeated-measures analysis of variance (ANOVA) was conducted for reaction time, accuracy and reaction time-accuracy ratio, respectively. For the ERP data, a 2 (session: HIIE vs. control) × 2 (congruency: congruent vs. incongruent) × 2 (picture type: high-calorie food picture vs. neutral picture) ANOVA was separately conducted for P3 and LPP average amplitudes from the Pz recording site, respectively. Follow-up comparisons were conducted with Bonferroni adjustment, effect sizes are presented as *η^2^_p_*, and statistical significance was set at *p* < 0.05.

## Results

### Behavioral data

**Fig. 2:**
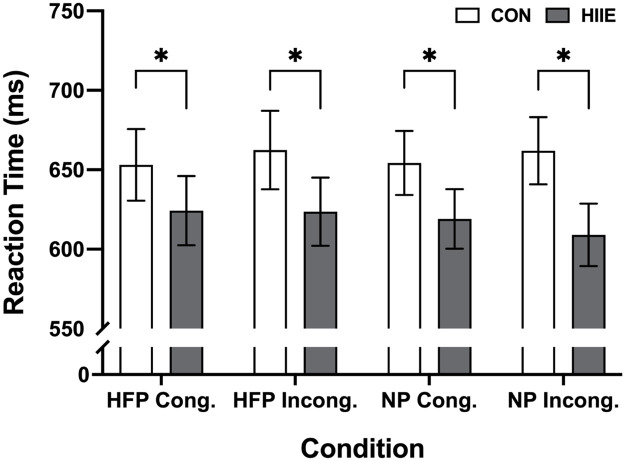
Reaction times for the food-related Flanker task by task congruency and picture types following high-intensity interval exercise and control sessions. Note. Values are means and standard errors. HIIE = High-intensity interval exercise; CON = Control; HFP = High-calorie food picture; NP = Neutral Picture. * *p* < 0.05.

Means and standard deviations for accuracy and reaction times by session, task congruency, and image type are reported in Table 2 and Fig. 2. The 2 (session: HIIE vs. control) × 2 (congruency: congruent vs. incongruent) × 2 (picture type: high-calorie food picture vs. neutral picture) repeated measures ANOVA for reaction time showed a significant main effect of session, *F* (1,14) = 5.94, *p* = 0.03, *η^2^_p_* = 0.30, with faster reaction time following the HIIE relative to the control session (619.00 ± 19.81 ms vs. 657.92 ± 21.72 ms). There was no credible evidence of significant main effect of task congruency [F (1,14) = 0.27, *p* = 0.61, *η^2^_p_* = 0.02] or picture type [*F* (1,14) = 0.62, *p* = 0.44, *η^2^_p_* = 0.04] or significant interaction between session and task congruency [*F* (1,14) = 4.24, *p* = 0.06, *η^2^_p_* = 0.23] or session and picture type [*F* (1,14) = 1.52, *p* = 0.24, *η^2^_p_* = 0.10] or congruency and picture type [*F* (1,14) = 1.46, *p* = 0.25, *η^2^_p_* = 0.09] nor an interaction among the three factors [*F* (1,14) = 0.41, *p* = 0.53, *η^2^_p_* = 0.03] for reaction time, *p* > 0.05, respectively*.*

**Table 2:** Reaction time (ms), accuracy (%), reaction time-accuracy ratio, P3, and LPP components for food and neutral pictures across task congruency for the food-related Flanker task following high-intensity interval exercise and control sessions.

Variable	HIIE session	Control session
RT (ms)		
HFP Congruent	624.27 ± 84.15	653.04 ± 87.19
HFP Incongruent	623.65 ± 83.03	662.38 ± 95.58
NP Congruent	619.05 ± 72.65	654.28 ± 77.99
NP Incongruent	609.04 ± 75.92	661.98 ± 81.86
Accuracy (%)		
HFP Congruent	92.10 ± 9.16	95.80 ± 4.25
HFP Incongruent	92.80 ± 9.91	96.20 ± 4.62
NP Congruent	92.73 ± 10.03	96.60 ± 3.58
NP Incongruent	93.93 ± 7.94	96.47 ± 4.22
Reaction time-accuracy ratio		
HFP Congruent	0.67 ± 0.13	0.68 ± 0.10
HFP Incongruent	0.68 ± 0.14	0.69 ± 0.13
NP Congruent	0.68 ± 0.13	0.68 ± 0.10
NP Incongruent	0.65 ± 0.11	0.69 ± 0.11
P3 (μV)		
HFP Congruent	2.07 ± 3.38	1.26 ± 2.55
HFP Incongruent	2.38 ± 3.17	1.32 ± 2.76
NP Congruent	2.73 ± 3.26	1.03 ± 2.60
NP Incongruent	2.08 ± 3.27	0.49 ± 3.18
LPP (μV)		
HFP Congruent	6.04 ± 4.10	1.55 ± 4.59
HFP Incongruent	5.32 ± 5.07	1.75 ± 4.52
NP Congruent	6.53 ± 3.68	1.79 ± 4.11
NP Incongruent	5.04 ± 4.82	1.68 ± 5.03

Note. HIIE = High-intensity interval exercise; CON = Control; RT= Reaction Time; HFP = High-calorie food picture; NP = Neutral Picture.

There was no credible evidence of significant main effect for session [F (1,14) = 3.01, *p* = 0.11, *η^2^_p_* = 0.18] or task congruency [F (1,14) = 1.30, *p* = 0.27, *η^2^_p_* = 0.09] or picture type [*F* (1,14) = 2.43, *p* = 0.14, *η^2^_p_* = 0.15] or interaction with session and task congruency [*F* (1,14) = 0.84, *p* = 0.38, *η^2^_p_* = 0.06] or session and picture type [*F* (1,14) = 0.36, *p* = 0.56, *η^2^_p_* = 0.03] or congruency and picture type [*F* (1,14) = 0.00, *p* = 1.00, *η^2^_p_* = 0.00] or interaction among the three factors [*F* (1,14) = 1.10, *p* = 0.31, *η^2^_p_* = 0.07] for accuracy, *p* > 0.05, respectively*.* Accuracy data for the food-related Flanker task are presented in Table 2.

The 2 (session: HIIE vs. control) × 2 (congruency: congruent vs. incongruent) × 2 (picture type: high-calorie food picture vs. neutral picture) repeated measures ANOVA for the reaction time-accuracy ratio showed a significant interaction among session, congruency and picture type [*F* (1,14) = 9.33, *p* = 0.01, *η^2^_p_* = 0.40]. Post hoc tests revealed a significant smaller reaction time-accuracy ratio for the neutral picture type relative to the high-calorie food picture type in incongruent condition following HIIE session (0.65 ± 0.11 vs. 0.68 ± 0.14, *p* = 0.03) and a significant smaller reaction time-accuracy ratio for the incongruent condition relative to the congruent condition in neutral picture type following HIIE session (0.65 ± 0.11 vs. 0.68 ± 0.13, *p* = 0.02). There is no credible evidence of significant main effect of session [*F* (1,14) = 0.29, *p* = 0.60, *η^2^_p_* = 0.02] or congruency [*F* (1,14) = 0.03, *p* = 0.87, *η^2^_p_* = 0.002] or picture type [*F* (1,14) = 0.76, *p* = 0.40, *η^2^_p_* = 0.05] or interactions between session and congruency [*F* (1,14) = 1.81, *p* = 0.20, *η^2^_p_* = 0.11], session and picture type [*F* (1,14) = 0.52, *p* = 0.48, *η^2^_p_* = 0.04] or congruency and picture type [*F* (1,14) = 2.20, *p* = 0.16, *η^2^_p_* = 0.14]. Reaction time-accuracy ratio data for the food-related Flanker task are presented in Table 2.

### EEG data

#### P3 component

**Fig. 3:**
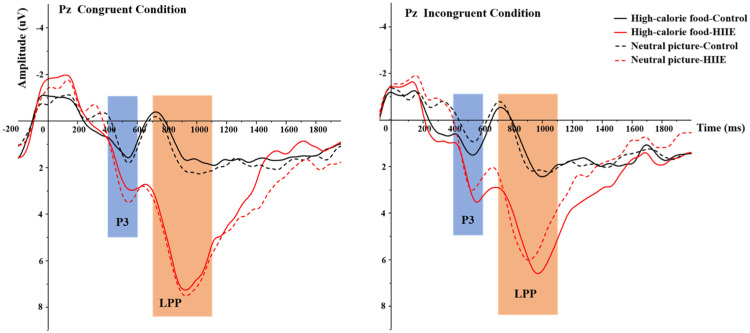
P3 (400-600 ms) and LPP (700-1100 ms) ERP component amplitudes for congruent task and incongruent conditions of the food-related Flanker task. *Note.* The solid black line represented the high-calorie food picture waveform in control session. The dotted black line represented the neutral picture waveform in control session. The solid red line represented the high-calorie food picture waveform in HIIE (high-intensity interval exercise) session. The dotted red line represented the neutral picture waveform in HIIE session. For congruent condition, P3 amplitude revealed no significant difference between the two sessions (*p* > 0.05, *p* = 0.06) or the two picture types (*p* > 0.05). LPP amplitude showed a significant larger amplitude following HIIE session compared with the control session (*p* = 0.01). No significant difference between the two picture types was found (*p* > 0.05). For incongruent condition, P3 amplitude revealed a significant larger amplitude for HIIE session to control session (*p* = 0.02). No significant difference between the two picture types was found (*p* > 0.05). LPP amplitude showed a significant larger amplitude following HIIE session compared with the control session (*p* = 0.03). No significant difference between the two picture types was found (*p* > 0.05).

P3 ERP waveforms by session, congruency, and image type are shown in Fig. 3. Means and standard deviations for P3 amplitude are reported in Table 2. The 2 (session: HIIE vs. control) × 2 (congruency: congruent vs. incongruent) × 2 (picture type: high-calorie food picture vs. neutral picture) repeated measures ANOVA for P3 amplitude revealed a significant main effect of session, *F* (1,14) = 5.63, *p* = 0.03, *η^2^_p_* = 0.29, with a larger amplitude observed following HIIE (2.31 ± 0.78 μV) compared with the control session (1.02 ± 0.68 μV). There was no credible evidence of significant main effect of task congruency [F (1,14) = 0.75, *p* = 0.40, *η^2^_p_* = 0.05] or picture type [*F* (1,14) = 0.95, *p* = 0.35, *η^2^_p_* = 0.06] or interaction between session and task congruency [*F* (1,14) = 0.09, *p* = 0.77, *η^2^_p_* = 0.01] or session and picture type [*F* (1,14) = 2.51, *p* = 0.14, *η^2^_p_* = 0.15] or congruency and picture type [F (1,14) = 1.34, p = 0.27, *η^2^_p_* = 0.09] or interaction among the three factors [F (1,14) = 0.15, p = 0.71, *η^2^_p_* = 0.01] for P3 amplitude, *p* > 0.05, respectively*.*

#### LPP component

LPP ERP waveforms by session, congruency, and image type are shown in Fig. 3. Means and standard deviations for LPP amplitude are reported in Table 2. The 2 (session: HIIE vs. control) × 2 (congruency: congruent vs. incongruent) × 2 (picture type: high-calorie food picture vs. neutral picture) repeated measures ANOVA for LPP amplitude revealed a significant main effect of session, *F* (1,14) = 8.77, *p* = 0.01, *η^2^_p_* = 0.39, with larger amplitude observed following HIIE (5.73 ± 1.00 μV) compared with the control session (1.69 ± 1.16 μV). There was no credible evidence of significant main effect of task congruency [F (1,14) = 1.61, *p* = 0.23, *η^2^_p_* = 0.10] or picture type [*F* (1,14) = 0.12, *p* = 0.73, *η^2^_p_* = 0.01] or interaction between session and task congruency [*F* (1,14) = 2.19, *p* = 0.16, *η^2^_p_* = 0.14] or session and picture type [*F* (1,14) = 0.00, *p* = 0.96, *η^2^_p_* = 0.00] or congruency and picture type [F (1,14) = 0.37, *p* = 0.55, *η^2^_p_* = 0.03] or interaction among the three factors [F (1,14) = 0.14, p = 0.72, *η^2^_p_* = 0.01] for LPP amplitude, *p* > 0.05, respectively*.*

## Discussion

The study investigated the effects and underlying neural mechanisms of a 30-min acute session of HIIE on food-related cognitive function and inhibitory control assessed by behavioral and ERP measures. This is the first known study to examine the influence of acute HIIE on food-related cognition and inhibitory control, as well as the P3 and LPP components elicited by a food-related cognitive paradigm among young adults with obesity. The key findings were that acute HIIE resulted in shorter response speed in the food-related Flanker task and increased P3 and LPP amplitudes, which indicate enhancement of general and food-related cognitive control as well as increased recruitment of cognitive resources to support cognitive control processes. Overall, these findings support the previous findings indicating that acute HIIE facilitates general cognitive function (Xie et al., 2020), and extent these findings to food-related cognitive control in young adults with obesity.

With respect to behavioral performance, response times following HIIE were faster than the control session, regardless of the congruency or picture type, accompanied with no credible evidence of a significant difference in accuracy. Previous studies have reported shorter reaction times in both the Flanker and Stroop tasks after similar HIIE interventions (Alves et al., 2014; Kao et al., 2017; Tian et al., 2023; Tsukamoto et al., 2016), which suggests that HIIE elicits enhanced speed of processing and facilitates efficiency in cognitive control (Kao et al., 2017). These results are also in accord with our earlier observations, which showed improvement in general cognitive control following an acute session of HIIE (Xie et al., 2020). The current findings extend this positive effect to food-related cognitive control in young adults with obesity. Similar to our findings, Bailey et al. (2021) found faster response times in a food-related Go/No Go task following an acute bout of aerobic exercise performed at 70% VO_2_max, which indicates improved and more efficient food-related cognitive control processes. It should be noted that we additionally analyzed the reaction time-accuracy ratio to examine the potential influence of the speed-accuracy trade-off. We found that there was no significant effect for the session, suggesting that the results might be influenced by the trade-off. Overall, while our findings showed that a single bout of HIIE facilitates both general and food-related cognitive control, the interpretation needs to be conservative.

Moreover, no difference was found in high-calorie food and neutral pictures, which indicates that high-calorie food images did not induce a larger effect than neutral objects. A number of studies have reported that obese individuals performed slower in high-calorie food than neutral stimulus cues in food-related dot-probe or Stroop tasks (Hagan et al., 2020; Husted et al., 2016). The improved reaction time does not relate specifically to the high-calorie food images after acute HIIE, potentially indicating that acute HIIE has general positive effect on cognitive processing and inhibitory control. Surprisingly, we also did not find a significant difference in congruent and incongruent trials, that is, no “conflict effect” (Eriksen & Eriksen, 1974) was observed. This may have resulted from our version of the Flanker task in that the flanking stimuli may not be resulting in the same level of conflict as the more traditional versions of the Flanker task (e.g., arrow version).

In terms of ERP evidence, acute HIIE induced a larger P3 amplitude than the control session, regardless of the picture type or task congruency. This result aligns with a number of previous studies that have observed an increase in P3 amplitude after acute exercise (Chang, Alderman, et al., 2017; Junyeon et al., 2017; Kao et al., 2022; Tsai et al., 2021), which suggests that a single bout of exercise increases attentional resource allocation to achieve successful goal-oriented behavior (Kao et al., 2022). However, it is important to note that the evidence for P3 amplitude changes after acute HIIE have not been consistent (Kao et al., 2018; Kao et al., 2017; Xie et al., 2020). For example, Kao et al. (2018) and Xie et al. (2020) did not observe any significant changes in Flanker P3 amplitude following HIIE, whereas (Kao et al., 2017) reported a smaller P3 amplitude to the Flanker task following acute HIIE. Kao and colleagues concluded that the reduced P3 amplitude following HIIE could be regarded as neural efficiency (Adam et al., 2012; Malinowski, 2013) due to the diminished recruitment of neural resources accompanied by enhanced cognitive control (Kao et al., 2017). Given the different P3-elicting tasks (classic Flanker task vs. food-related Flanker task), exercise duration (9-min vs. 16-min vs. 30-min) and the P3 time windows as well as the electrode site used (300-400 ms at Fz site vs. 400 to 600 ms at Pz site), discrepancies in findings may be explained by key exercise characteristics, nature of the P3-elicting tasks, and ERP data collection and analysis details. Considering the enlarged P3 amplitude accompanied by more efficient reaction time performance, the current results suggest that an acute HIIE facilitates general and food-related cognitive processes, and neural resource allocation as indicated by overall enhanced P3 amplitude.

Similar to the P3, an increase LPP amplitude was observed following HIIE. LPP is a late potential which reflects higher-order attentional processes (Carbine et al., 2018; Schupp et al., 2003) and arousal level as well as precise processing of stimuli (Benau et al., 2019; Shestyuk & Deldin, 2010). Potentiated LPP amplitude suggests increased allocation of attention in stimulus processing (Carbine et al., 2018; Schupp et al., 2003) and cognitive control (Carbine et al., 2018; Watson & Garvey, 2013). Though few studies have investigated LPP component amplitude after acute exercise intervention, some studies have reported enhanced LPP amplitude following an acute aerobic exercise, which indicates improved reactivity to the target stimulus (Brush et al., 2021; Brush et al., 2020). Additionally, our pervious study showed increased LPP amplitude after a single bout of HIIE, suggesting an increased capacity of attention allocation and inhibition of irrelevant stimuli in support of cognitive inhibition (Xie et al., 2020). Collectively, these findings suggest that acute HIIE results in an increase of attention allocation to facilitate general and food-related cognitive processes.

The major strength of this study is the examination of the acute HIIE effects and the ERP mechanisms on general and food-related cognition in young adults with obesity. However, this study has several limitations. First, this study included a relatively small sample size, and therefore future studies incorporating larger samples are warranted. Additionally, we restricted this initial study to male participants given that previous studies have found sex differences in food-related craving, cognitive function, and neural responses to food-related stimuli (Hallam et al., 2016; Ravichandran et al., 2021). Studies examining neural responses to food-related cognitive tasks by sex, and the influence of exercise and HIIE on food-related cognition are needed. It may also be useful to examine exercise effects on women across phases of the menstrual cycle, as this may potentially influence both exercise responses and acute influences on cognition. Third, variables like nutrition status and sedentary behavior have the potential to impact exercise-induced changes in food-related cognition. In future studies, examination of these variables is warranted, and it is advisable to include nutritional status, sedentary behavior, dietary patterns, intelligence and additional factors as covariates in studies incorporating larger sample sizes. Furthermore, it's essential to acknowledge that our results might be influenced by speed-accuracy trade-offs. Hence, forthcoming studies should meticulously address this trade-off issue while prudently replicating and expanding upon the findings of our research. Lastly, in addition to the specific mode of exercise (HIIE versus more traditional moderate-intensity aerobic exercise), there is a need to examine dose-response relationships between acute or long-term HIIE and food-related cognition in order to guide exercise prescriptions for improving upstream targets for obesity (e.g., food-related cognition).

## Conclusion

A single bout of HIIE results in a beneficial effect on general and food-related cognition among young adult males with obesity. Based on the P3 and LPP components, the underlying cognitive mechanisms may include an increased recruitment of neural resources to support cognitive and inhibitory control. Future studies encourage to include larger sample sizes and both men and women, and it is recommended to further examine the dose-response relationship between acute or long-term HIIE and food-related cognition in order to guide more precise exercise prescriptions for the prevention and treatment of obesity.

## Funding

This study was funded by grants from the National Social Science Foundation (No. 21BTY030) to K.W.

## Data availability

The data presented in this study are available on request from the corresponding author.

## CRediT authorship contribution statement

**Chun Xie:** Conceptualization, Methodology, Formal analysis, Writing – original draft, Writing – review & editing. **Brandon L. Alderman:** Formal analysis, Investigation, Writing – original draft, Writing – review & editing. **Fanying Meng:** Conceptualization, Investigation, Writing – original draft, Writing – review & editing. **Ying-Chu Chen:** Methodology, Investigation, Writing – original draft, Writing – review & editing. **Yu-Kai Chang:** Conceptualization, Methodology, Formal analysis, Writing – original draft, Writing – review & editing. **Kun Wang:** Conceptualization, Methodology, Investigation, Writing – original draft, Writing – review & editing.

## Declaration of Competing Interest

The authors declare that they have no known competing financial interests or personal relationships that could have appeared to influence the work reported in this paper.

## Figures and Tables

**Figure:**
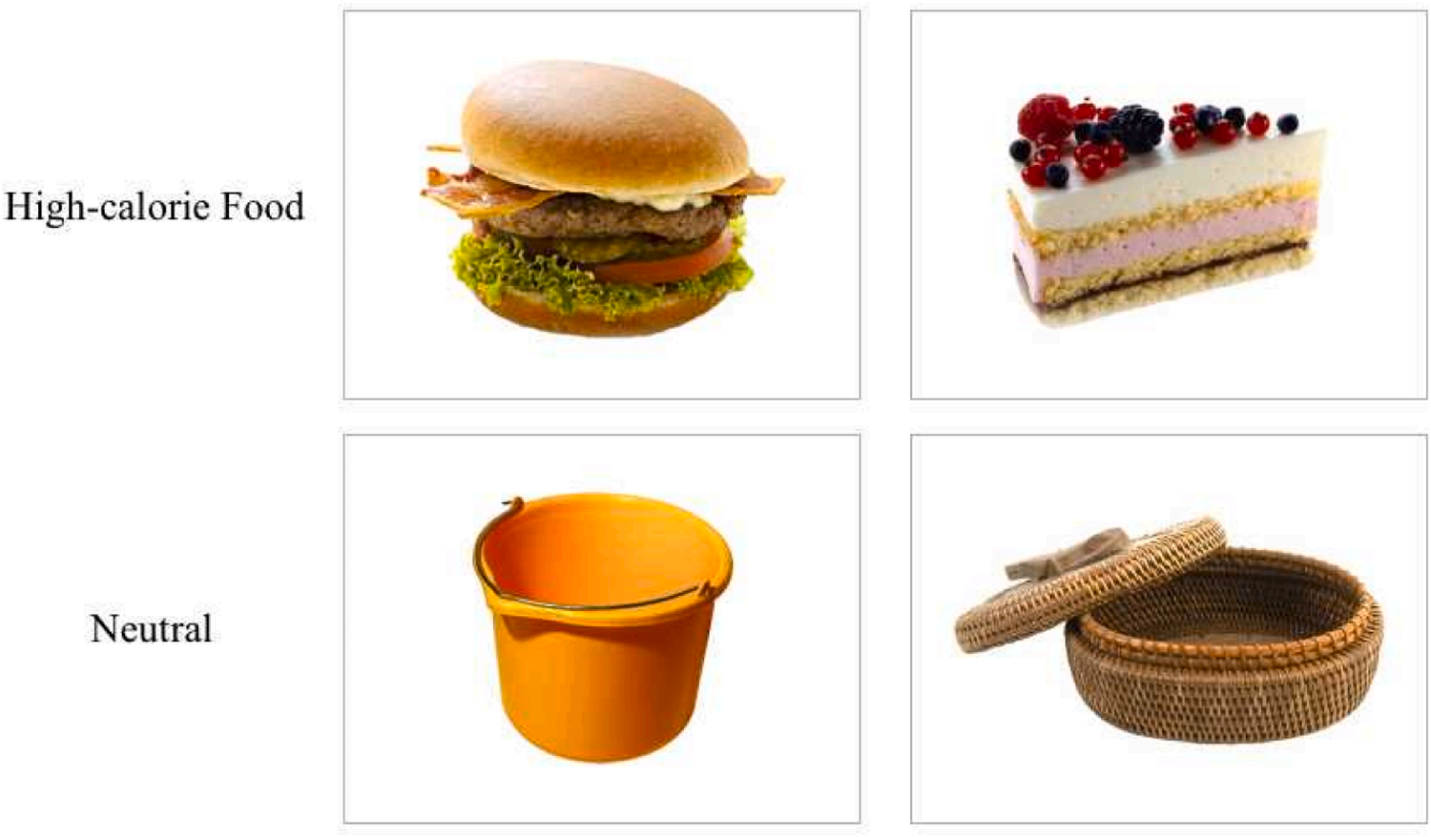


**Table:** 

Picture Type	Food-pics Database Number
High-calorie food	1, 2, 6, 12, 22, 23, 34, 41, 42, 43, 45, 46, 53, 56, 57, 60, 69, 77, 79, 80, 86, 87, 89, 91, 103, 105, 115, 127, 136, 137, 140, 148, 151, 159, 167, 169, 171, 176, 185, 287, 295, 296, 297, 298, 309, 310, 313, 318, 319, 363, 387, 469, 485, 489, 507, 517, 533, 538, 547, 555
Neutral	1004, 1005, 1007, 1008, 1009, 1010, 1013, 1014, 1028, 1030, 1031, 1032, 1033, 1035, 1038, 1056, 1060, 1134, 1140, 1141, 1142, 1143, 1146, 1149, 1151, 1153, 1156, 1208, 1210, 1212, 1213, 1214, 1218, 1219, 1223, 1224, 1225, 1227, 1231, 1234, 1235, 1239, 1240, 1242, 1243, 1244, 1249, 1250, 1252, 1256, 1258, 1261, 1265, 1267, 1268, 1272, 1274, 1276, 1277, 1279

## Appendix A

Examples of the high-calorie food and neutral pictures.

## Appendix B

Pictures employed in the experiment from the food-pics database (Blechert et al., 2014).
